# Mcl-1 inhibitor suppresses tumor growth of esophageal squamous cell carcinoma in a mouse model

**DOI:** 10.18632/oncotarget.18772

**Published:** 2017-06-28

**Authors:** Jianqing Lin, Deqiang Fu, Yijun Dai, Jianguang Lin, Tianwen Xu

**Affiliations:** ^1^ Department of Medical Oncology, The Second Affiliated Hospital of Fujian Medical University, Quanzhou, Fujian, China

**Keywords:** ESCC, Mcl-1, A-1210477

## Abstract

Esophageal squamous cell carcinoma (ESCC) has a high morbidity in China, accounting for 90% of all esophageal carcinoma cases. Hence, identifying drug targets for prevention and treatment of ESCC is essential. Due to its critical role in the regulation of cell apoptosis, Mcl-1 holds great potential as a target for treatment against ESCC. In current study, we used a 4-nitroquinoline-1-oxide (4-NQO)-induced ESCC mouse model of test whether A-1210477, a Mcl-1 small molecular inhibitor, could repress ESCC development. We showed that A-1210477 treatment decreased ESCC formation and animal weight loss in a dose dependent manner. We detected decreased cellular proliferation in A-1210477-treated ESCC tissue by Ki67 expression. Moreover, A-1210477 treatment increased the number of apoptotic cells in ESCC tissues. Our study clearly demonstrates the contribution of Mcl-1 to ESCC development through promoting cell proliferation and inhibition of apoptosis, and provides a strong evidence for further evaluation of A-1210477 for treating ESCC.

## INTRODUCTION

Esophageal squamous cell carcinoma (ESCC) is one of the most usual malignancies, ranked as the sixth leading cause of death from cancers internationally [1]. China has the highest incidence and death rate of ESCC in the world [1, 2]. Current treatments for ESCC include surgery, chemotherapy and radiation therapy. However, the ESCC cancer mortality remains over 60% due to recurrence, metastasis, drug resistance and advanced disease [2]. Because of the strikingly poor prognosis, developing novel and more effective strategies for ESCC treatment is urgently needed.

Myeloid leukemia-1 (Mcl-1) is an anti-apoptotic factor, which belongs to the Bcl-2 family. Mcl-1 is overexpressed in a variety of cancerous tissues and is the cause of resistance to common chemotherapies [3]. The cellular expression of Mcl-1 is tightly regulated via multiple mechanisms [4]. Studies have shown that distinct signaling pathways are involved in the activation of Mcl-1 gene expression in different tumor cells, including mitogen-activated protein kinase (MAPK), phosphatidylinositol 3-kinase (PI-3K) and JAK/STAT signal [4]. In human ESCC, NF-κB subunits p50 and p65 can bind to Mcl-1 promoter and activate the expression of Mcl-1 [5]. In addition, p-Stat3 and Mcl-1 are found to be overexpressed in ESCC tissues and contribute to apoptotic resistance in ESCC cells [6]. Blocking of MAPK, PI-3K and NF-κB pathways can cause apoptosis and reduce the expression of Mcl-1, suggesting that Mcl-1 as a new target for clinical anti-ESCC therapy.

A-1210477 is small molecule compound that can trigger the intrinsic apoptosis of multiple cancer cell lines [7, 8]. A-1210477 binds to MCL-1 with high affinity and induces MCL-1 protein elevation in cells [7, 8]. The aim of current study is to develop novel strategies to efficiently kill ESCC tumor cells by utilizing recently developed Mcl-1 inhibitor A-1210477 that directly activate the cell death pathway. We found that treatment of A-1210477 dramatically reduced ESCC tumor burden in a chemically induced mouse model. A-1210477 functioned as an anti-proliferation and pro-apoptosis factor in mouse ESCC. Hence, our study has important clinical implications with regard to development and prevention of ESCC.

## RESULTS

### NFkB and Mcl-1 are activated in 4NQO-induced ESCC

**Figure 1:**
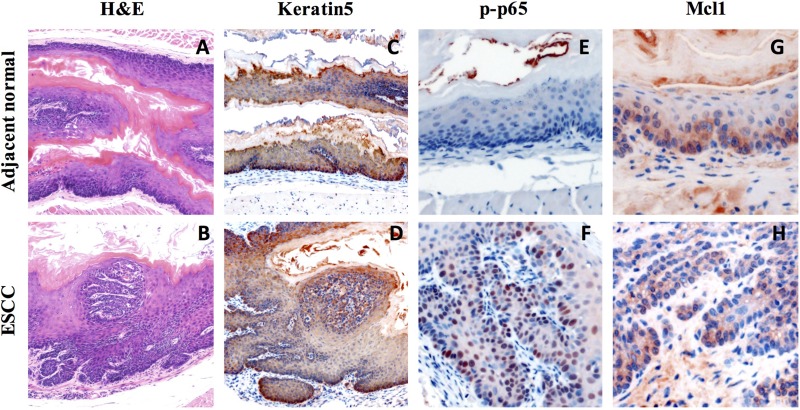
Increase expression of inflammation marker and Mcl-1 in 4NQO-induced mouse ESCC (**A–B**) Histological analysis of 4-NQO-induced lesions in the mouse esophagus by H&E staining; (**C–D**) Immunohistochemical analysis of basal cell marker (Keratin5) expression in 4-NQO-induced tumor; (**E–F**) Immunohistochemical analysis of inflammation marker (p-p65) in 4-NQO-induced tumor; (**G–H**) Immunohistochemical analysis of Mcl-1 expression in 4-NQO-induced tumor.

Our H&E staining revealed formation of ESCC in all 4NQO-treated mice harvested at end point (Figure 1A, 1B). The expression of basal cells K5 was detected in the epithelium of both normal adjacent tissue and ESCC lesion area (Figure 1C, 1D). We also observed elevated NFκB activation by p-p65 IHC staining (Figure 1E, 1F), indicating a link between inflammation and ESCC formation. To investigate the role of Mcl-1 in the development of mouse ESCC, we examined Mcl-1 expression and activity and fount that Mcl-1 is highly expressed in both normal and ESCC tissues (Figure 1G, 1H).

### A-1210477 suppressed ESCC formation in mice

**Figure 2:**
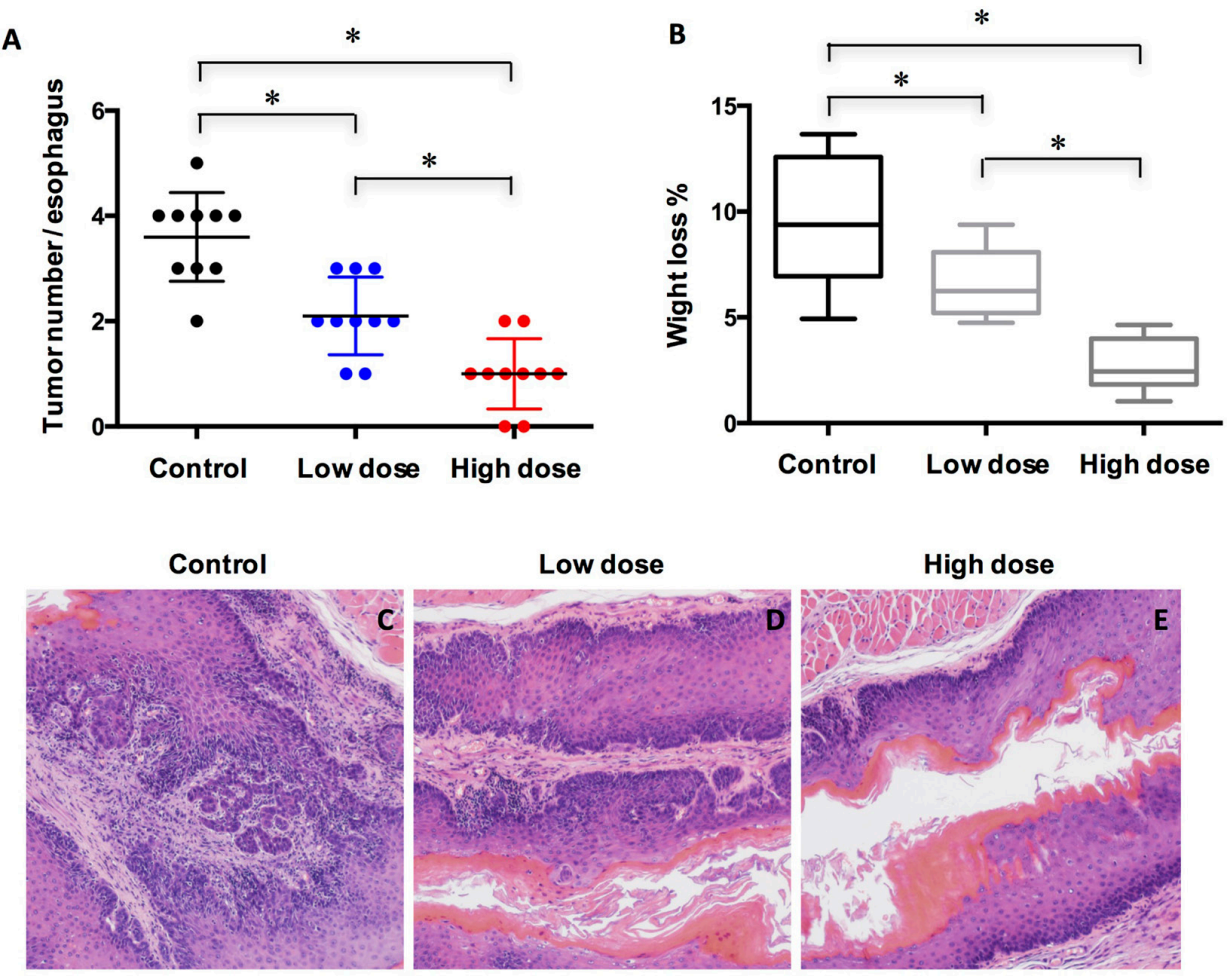
Treatment of A-1210477 inhibits ESCC formation in mice (**A**) Quantified results showed the A-1210477 reduced the incidence of 4NQO-induced ESCC in a dose-dependent manner; (**B**) Quantification of the body weight loss showed A-1210477 inhibited weight loss in a dose-dependent manner; (**C**–**E**) Histological analysis of ESCC treated with A-1210477 by H&E staining. The data are presented as mean ± SD. Tumor incidence and weight loss differences were analyzed by one-way ANOVA. ^*^P < 0.05.

Based on the expression of Mcl-1 in ESCC tissue sample, we reasoned that Mc1-1 might play a role in ESCC cancer progression. To test our hypothesis, mice at 20 weeks after initial 4NQO exposure were injected with control vehicle, lose dose and high dose of A-1210477 daily for 4 weeks. Our results showed that A-1210477-treated mice had developed fewer tumors than the vehicle-treated mice did in a dose dependent manner (Figure 2A). Similarly, there was significant less body weight loss in the A-1210477-treatment group mice compared with the control ones (Figure 2B). Microscopically, we also noticed less malignancy formation in the esophagus following A-1210477-treatment (Figure 2C–2E).

### A-1210477 inhibited cell proliferation and promoted cell death in ESCC

**Figure 3:**
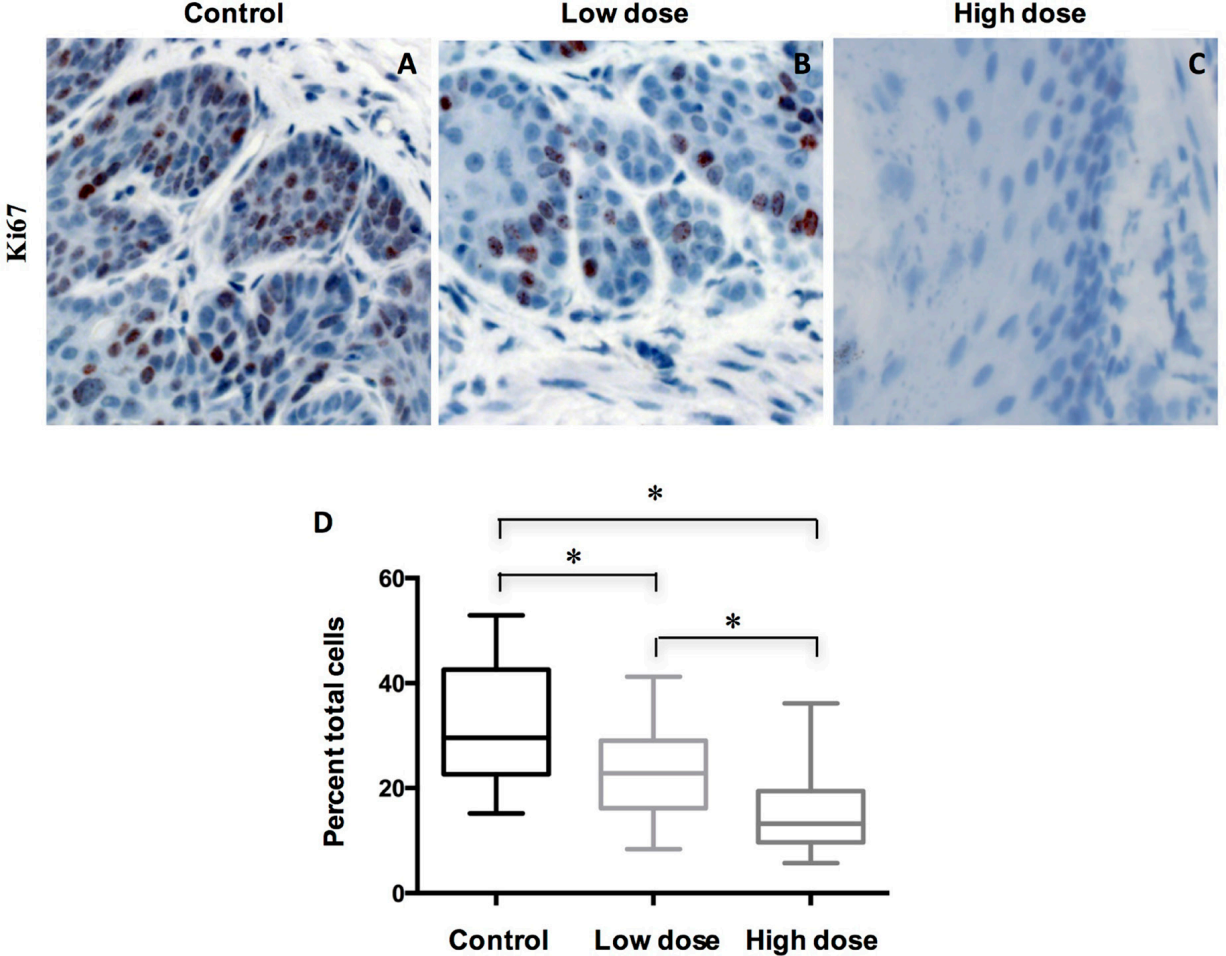
Treatment of A-1210477 decreased cell proliferation in mouse ESCC (**A**–**C**) Tissue sections of ESCC treated with different conditions were immunohistochemically stained for Ki-67; (**D**) Ki67 labeling indices were quantified in different treatment groups. The data are presented as mean ± SD. The difference was analyzed by one-way ANOVA. ^*^P < 0.05.

To understand how A-1210477 inhibited ESCC tumor progression, we examined the cell proliferation by Ki67 IHC staining. Our data showed that there was a statistically significant difference (*P* < 0.05 by ANOVA) in Ki67 labeling indices when control group (mean ± SD: 32.3 ± 11.9) was compared with groups with low dose (22.4 ± 9.0) and high dose of A-1210477 (15.0 ± 7.2) (Figure 3). Thus, esophageal cell proliferation was reduced markedly following A-1210477 treatment.

**Figure 4:**
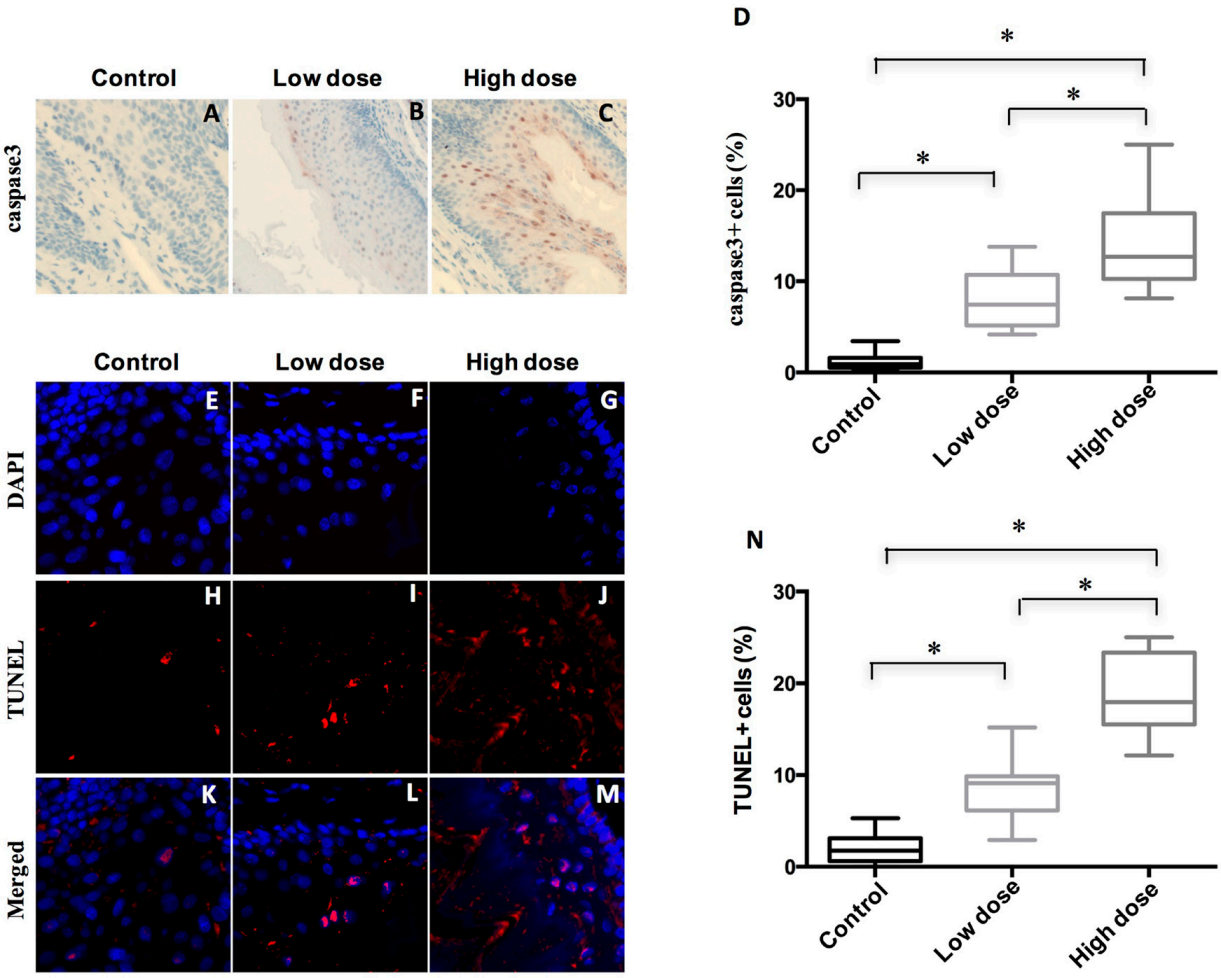
Treatment of A-1210477 promoted cell death in mouse ESCC (**A**–**C**) Immunohistochemical analysis of cleaved caspase3 to evaluate tumor cell apoptosis in mouse esophagus after A-1210477 treatment. (**D**) Quantitative analysis of A-1210477-induced cleaved caspase3 positive cells in mouse ESCC. (**E**–**M**) A-1210477 induced apoptosis detected by TUNEL staining in mouse ESCC. TUNEL (red) and DAPI (blue). (**N**) Quantitative analysis of A-1210477-induced TUNEL positive cells in mouse ESCC. The data are presented as mean ± SD. The difference was analyzed by one-way ANOVA. ^*^P < 0.05.

To further probe how A-1210477 inhibited ESCC tumor progression, we evaluated the apoptotic cells of these ESCC after A-1210477 treatment by detecting cleaved caspase3 expression with quantitative IHC. Our data demonstrated that there was a statistically significant increase in labeling indices when control ESCC (1.2 ± 0.8) was compared with low dose (8.0 ± 3.1) and high dose ones (14.0 ± 4.5) (Figure 4A–4D). To further validate our results, we analyzed apoptotic cells with TUNEL. The percentage of TUNEL positive cells increased dose dependently from 1.9 % in DMSO mice to 8.9 % (*p* < 0.05) in low-dose A-1210477 mice and 19.0 % (*p* < 0.05) in high-dose A-1210477 mice (Figure 4E–4N). Therefore, our data clearly demonstrated A-1210477 treatment led to increased cell death in mouse ESCC.

## DISCUSSION

ESCC is the predominant subtype of esophageal cancer, with a 5-year survival of only 10%. Thus, insights into developing novel treatment strategies are critical. Mcl-1 is overexpressed in many cancers and has been a very attractive drug target for treating cancer. Mcl-1 small molecular inhibitors, such as A-1210477 and S63845, have been proved to potently eliminate various kinds of Mcl1-dependent cancer cells [3, 9]. However, whether Mcl-1 inhibitor can repress ESCC development remains undefined. In current study, we used 4NQO-induced mouse ESCC model to test the function of Mcl-1 inhibitor A-1210477 *in vivo*. We showed that A-1210477 could release mouse tumor burden in a dose dependent manner. Specifically, A-1210477 can repress the cell proliferation and increase cell death of ESCC in mouse. Our findings have implications for therapeutic approach in treating ESCC.

Previous reports have shown that the expression of NF-kB p65 in ESCC tissues was positively associated with clinical staging, lymph node metastasis and tumor differentiation [10, 11]. Importantly, Mcl-1 expression in human ESCC is under control of the activation of NFκB signaling [5]. In our 4NQO-induced ESCC mouse model, we detected elevated level of p-p65 in the ESCC tissue compared to normal tissue control, suggesting that p-p65 might play a role in ESCC progression. Our data showed high expression level of Mcl-1 in ESCC tissue. However, whether the expression of Mcl-1 depends on NFκB signaling in mouse model needs to be more rigidly tested.

Mcl-1 plays a critical role in the survival of multiple cell types and is one of the highly amplified genes in cancer [12]. Recently, a group showed a novel Mcl-1 inhibitor S63845, which inhibits Mcl-1 via binding to the BH3-binding groove of Mcl-1, was able to potently kill various cancer cells both *in vitro* and *in vivo* [9]. Mechanistically, S63845 can cause cell death through the BAX/BAK-dependent mitochondrial apoptotic pathway [9]. This study provides evidence that inhibition of Mcl-1 might be a common therapeutic method for treating cancers. We showed here that inhibition of Mcl-1 by A-1210477 caused ESCC repression. However, whether S63845 can be used to target ESCC in mouse model remains unknown. It is known that Mcl-1 functions as a pro-survival faction via its interaction with Bak and Bim. On the other hand, Bak or Bim results in apoptosis if release from Mcl-1 [13]. Furthermore, binding of Mcl-1 to NOXA protein triggers degradation of Mcl1followed by caspase activation. In contrast, suppression of NOXA cause inhibition of apoptosis [14]. The exact molecular mechanism how A-1210477 induces the apoptosis of ESCC cells needs further investigation.

In conclusion, our studies clearly demonstrate a role of A-1210477 in ESCC inhibition. The outcomes of this study also shed light on understanding Mcl-1 in ESCC tumor development and suggest that Mcl-1 can be a target for preventing ESCC development.

## MATERIALS AND METHODS

### Mice

The protocol was approved by the Institutional Laboratory Animal Care and Use Committee of the Second Affiliated Hospital of Fujian Medical University, Fujian, China. For study of A-1210477 in ESCC development, a total of 45 eight-week-old female C57BL/6 mice were obtained from Model Animal Research Center of Nanjing University and randomly divided into control (*n* = 15), low dose (*n* = 15) and high dose (*n* = 15) groups. For induction of ESCC, mice were provided with 100 mg/mL 4-Nitroquinoline N-oxide (Meryer Chemical Technology, Shanghai, China) for 16 weeks and then fed with normal water for additional 8 weeks. Mcl-1 small chemical inhibitor A-1210477 (Selleckchem, China) was dissolved in 10% DMSO and 90% saline. To study the effect of A-1210477 on ESCC development, mice at 20 weeks of initial 4NQO exposure were subcutaneously injected with vehicle control, low dose A-1210477 (5 mg/kg) or high dose A-1210477 (20 mg/kg) for 4 weeks. The mice were weighted at 20 weeks and 24 weeks of initial 4NQO treatment. The weight loss was calculated by the weight difference between 20 weeks and 24 weeks, divided by the weight at 20 weeks and multiplied by 100. The mice were euthanized and esophagus tissues were harvested for characteristics analysis of tumor formation.

### Histology and immunohistochemistry (IHC) analysis

The esophagus tissues were fixed in 10% buffered formalin for 48 hours. The paraffin-embedded 5 μm-thick esophagus tissue sections from different groups were processed for H&E staining. For IHC staining, slides were incubated at 55°C for 2 hours and deparaffinized in xylene and rehydrated through graded alcohols (100%, 95%, 70% alcohol; 2 times 3 minutes in each grade). Slides were rinsed in ddH_2_O and then blocked with 0.3% H_2_O_2_ for 30 minutes to block endogenous peroxidase activity. Heat-induced antigen retrieval was performed in sodium citrate buffer (10 mM sodium citrate, 0.05% Tween 20, pH 6.0) using a pressure cooker. Sections were rinsed in PBS and blocked in 10% normal donkey serum, 1% bovine serum albumin and 0.1% Triton X-100. Sections were incubated with primary antibody against Keratin 5 (Abcam, ab52635, 1:500), Mcl1 (Abcam, ab32087, 1:200), p-p65 (Abcam, ab86299, 1:100), caspase3 (Cell Signaling, #9663, 1:500) and Ki67 (Thermo Fisher Scientific, RM-9106-S1, 1:200) overnight at 4°C. Section were rinsed in PBS and incubated with biotinylated secondary antibody and streptavidin-biotinylated horseradish peroxidase complex (Zhongshan, Beijing, China) 1 hour at room temperature. Proteins expression was detected by using Diaminiobenzidine (DAB) as substrate.

### TUNEL assay

Cell apoptosis was assayed by TUNEL staining using the TMR-Red Kit according to the manufacturer’s instructions (Roche). Cryosections from esophagus tissue were air-dried at room temperature for 10 min, fixed with 4% paraformaldehyde in PBS (pH 7.4) for 5 min, permeabilized with 0.1% Triton X-100 for 1 min, and incubated TUNEL reaction mixture in a humidified chamber at 37°C for 1 h in the dark. Sections were then counterstained with DAPI. We calculated the percentage of TUNEL positive cells by dividing the number of TUNEL-positive nuclei by the total number of DAPI-positive nuclei and multiplied by 100.

### Statistical analysis

Data are presented as the mean ± standard deviation (SD) of three independent experiments. Statistical analysis was performed using IBM SPSS Statistics. Differences between three groups were analyzed using a with one-way ANOVA followed by a post-hoc Tukey test; *p* values < 0.05 was considered statistically significant.
